# Neurosurgical CSF Diversion in Idiopathic Intracranial Hypertension: A Narrative Review

**DOI:** 10.3390/life11050393

**Published:** 2021-04-26

**Authors:** Geraint J. Sunderland, Michael D. Jenkinson, Elizabeth J. Conroy, Carrol Gamble, Conor L. Mallucci

**Affiliations:** 1 Department of Paediatric Neurosurgery, Alder Hey Children’s Hospital NHS Foundation Trust, Liverpool L12 2AP, UK; conor.mallucci@alderhey.nhs.uk; 2 Department of Neurosurgery, The Walton Centre NHS Foundation Trust, Liverpool L9 7LJ, UK; jenkinmd@liverpool.ac.uk; 3 Institute of Systems, Molecular and Integrative Biology, University of Liverpool, Liverpool L69 7BE, UK; 4 Liverpool Clinical Trials Centre, University of Liverpool, Liverpool, L69 3GL, UK; ejconroy@liverpool.ac.uk (E.J.C.); carrolp@liverpool.ac.uk (C.G.); 5 Faculty of Health and Life Sciences, University of Liverpool, Liverpool L69 7TX, UK

**Keywords:** idiopathic intracranial hypertension, pseudotumour cerebri, cerebrospinal fluid, ventriculoperitoneal shunt, lumboperitoneal shunt, programmable valve, anti-siphon device, neurosurgery

## Abstract

The prevalence of idiopathic intracranial hypertension (IIH), a complex disorder, is increasing globally in association with obesity. The IIH syndrome occurs as the result of elevated intracranial pressure, which can cause permanent visual impairment and loss if not adequately managed. CSF diversion via ventriculoperitoneal and lumboperitoneal shunts is a well-established strategy to protect vision in medically refractory cases. Success of CSF diversion is compromised by high rates of complication; including over-drainage, obstruction, and infection. This review outlines currently used techniques and technologies in the management of IIH. Neurosurgical CSF diversion is a vital component of the multidisciplinary management of IIH.

## 1. Introduction

Idiopathic intracranial hypertension (IIH) is a rare disorder affecting approximately 0.9–3/100,000 of adults [1,2,3]. This incidence is estimated to have more than tripled in the last 30 years in association with rising rates of obesity [4,5]. Females of childbearing age with body mass indexes (BMI) in the obese range are at particular risk, with incidence up to 20 times that of the general population [6]. IIH was first described by Quincke in 1893 and further outlined by Dandy in 1937 as a syndrome where patients present with symptoms and signs of raised intracranial pressure but without any evidence of mass lesion [7,8]. IIH is characterised by headaches of variable phenotype, vomiting, and visual disturbance (blurred vision, transient obscurations, and visual field loss), in association with raised intracranial pressure (ICP) [9]. Less frequently associated symptoms include pulsatile tinnitus and diplopia [10]. Elevated ICP, while not life-threatening, is often sight-threatening, and as many as 30% of sufferers progress to permanent visual loss [2,11].

Symptomatically, IIH is characterised as a headache syndrome that has significant crossover with migraine disorders, often existing in conjunction [12]. It typically exists in the absence of other neurological deficits, with the exception of sixth cranial nerve palsy [13]. From an epidemiological standpoint, IIH demonstrates a strong predilection for females of child-bearing age, in particular, an association with high BMI. The most effective treatment approach is weight management in typical cases; 15% weight loss has been shown to be associated with resolution of raised ICP [14].

The underlying aetiology of IIH remains obscure; however, interplay between BMI, raised cranial venous pressure and/or outflow obstruction, and resistance to CSF resorption is the most popular hypothesis [15]. The common endpoint to this pathway is resistance to CSF reabsorption via arachnoid granulations and through extracranial lymphatics [16,17].

IIH continues to present unique challenges to clinicians involved in its management. It is a truly multi-disciplinary condition requiring coordinated input from neurology, ophthalmology, optometry and neurosurgery. For the majority of patients, non-surgical treatments that include medication and weight management suffice in controlling the disease; however, up to a quarter of IIH patients require surgical or interventional radiology treatment in response to visual deterioration [18].

While headache associated morbidity is significant, the threat to vision presents the most alarming consequence of inadequately managed IIH [19]. Fortunately, effective management of ICP can eliminate the risk of visual loss—a direct consequence of pressure on the retinal nerve fibre layer at the optic disc [20].

CSF diversion constitutes the mainstay of neurosurgical management of IIH. The purpose of this article is to review the practice of neurosurgical CSF diversion in CSF and to outline a ‘what-to-do-when’ algorithm for suspected shunt failure.

## 2. Materials and Methods

We performed a comprehensive literature search on the electronic database MEDLINE (PubMed) to identify original research articles and reviews on the neurosurgical management of IIH. A further literature search for published systematic reviews and meta-analyses were performed using MEDLINE (PubMed) and the Cochrane database. Databases were searched using relevant combinations of appropriate keywords, including “idiopathic intracranial hypertension”, “pseudotumor cerebri”, “benign intracranial hypertension”, “CSF diversion”, “ventriculoperitoneal”, “lumboperitoneal”, and “shunt”. Additional studies were found by reviewing the reference lists of the identified publications. A narrative review was produced based on these searches.

## 3. Results

### 3.1. Neurosurgical Involvement in IIH

Neurosurgeons have a very defined role in the management of IIH, and are consulted when there is evidence of papilloedema or visual deficits that are progressive [12,21]. In the majority of circumstances, patients will present with headache symptoms primarily; visual sequelae of papilloedema are more commonly transient and self-resolving in the initial stages. Untreated papilloedema can cause progressive and permanent visual loss with optic atrophy; this will occur in up to 30% of IIH patients [19,22]. Evidence of rapid progression of visual loss within 4 weeks of diagnosis indicates a fulminant form of IIH and urgent intervention is required to prevent disabling permanent visual loss [23]. In the context of stable papilloedema and visual function, patients will undergo a trial of non-surgical management, including weight loss in combination with medication (most commonly, acetazolamide) [9,12]. Consideration of surgical intervention is indicated in cases that prove refractory to conservative measures [21]. Progression of papilloedema and visual deficit is an absolute indication for surgical intervention. Intolerance of acetazolamide is also common due to its significant side effect profile and surgical intervention is occasionally indicated in such cases [24].

#### 3.1.1. CSF Diversion Strategies

Historically, a variety of cranial vault expansion and decompression techniques have been applied to the management of IIH. In particular, subtemporal decompression (STD), which is performed via either unilateral or bilateral temporal craniectomy, found some broad application [7,25]. The development and expansion of internalised CSF diversion systems, which have since become the central surgical strategy in IIH management, obviate the need for such surgeries, although STD is still performed in exceptional circumstances [26,27]. CSF diversion may be in the form of lumboperitoneal (LPS) or ventriculoperitoneal shunting (VPS). The primary aim of surgical intervention is preservation of visual function [21]. While the substantial headache morbidity is seen to improve in many cases, this is not as reliable or sustained, and so, is not the metric by which intervention success is judged [6,28]. Surgical intervention, particularly lumboperitoneal shunting, can in fact compound headache symptoms due to CSF over-drainage and the development of low pressure symptoms [29].

#### 3.1.2. LP Shunt

The practice of lumboperitoneal shunting was historically the preferred CSF diversion technique as the ventricles in IIH patients are typically small with attendant technical challenges [30,31]. Accessing CSF in the lumbar cistern bypasses this challenge, but accessing the spinal canal can present its own technical difficulties, especially in those with obese body habitus. Likewise, obtaining safe access to the peritoneum with the obese patient in the lateral position can be difficult and repositioning mid-surgery may be required.

The catheter can be sited within the lumbar subarachnoid cistern via a lumbar puncture or under direct vision via an open incision. This catheter is anchored and connected in line with a valve and distal tubing tunnelled to the abdomen. In very rare circumstances, the pleural cavity has also been described as an alternative CSF resorption site [32].

LP shunts have demonstrated efficacy in improving visual symptoms in 55–100% of cases reported and headache symptoms are likewise responsive, up to 76–92% cases report improvement, at least initially [31,33,34,35].

LP shunts are prone to complications, more so than VP shunts. Up to 40% of patients will require shunt revision, with many requiring multiple revisions; median revision per patient rate = 4.3 in a recent systematic review compared with 1.83 for VP shunts [18]. Over-drainage complications are frequently seen with development of secondary Chiari malformation (seen in up to 30% of patients) [36]. Mechanical failure is common in LP shunts, as is catheter migration and ‘flipping’ of the valve, which is difficult to anchor in the subcutaneous tissues. This presents specific difficulties when attempting to adjust programmable valves, which require precise alignment of percutaneous programming devices. In recent years, LP shunts have thus seen a drop in popularity with surgeons preferring to install VP shunts, due in large part to the lower revision rate [28,30,37,38].

#### 3.1.3. Cranial CSF Shunts

Insertion of VPS is the most common CSF diversion procedure performed for IIH nowadays and is proven effective [31,37,39,40,41,42,43]. Efficacy in arresting visual failure, and in many cases reversing visual deficit, has been demonstrated in 55–89% of patients [18]. Headache symptoms are also seen to improve at least initially; however, they commonly return or the headache syndrome may be complicated by low-pressure symptoms [6,28].

Under general anaesthetic, a ventricular catheter (thin silicon catheter) is advanced into the ventricle, most commonly the right lateral ventricle. Use of antibiotic (rifampicin + clindamycin) impregnated Bactiseal® catheters to reduce the risk of shunt infection is supported by level 1 evidence [44]. This catheter is connected to a valve that regulates CSF egress based either on a differential pressure gradient or, less commonly, a constant flow rate. There is a multitude of valve designs and features; the most common of which will be discussed later in this article. The valve is, in turn, connected to a distal length of a catheter, which is tunnelled subcutaneously to the abdomen and peritoneal cavity where CSF is reabsorbed. The cardiac atria via superior vena cava or the pleural cavity present alternative sites of distal implantation, in the rare cases when the peritoneal cavity is not suitable.

**Figure 1:**
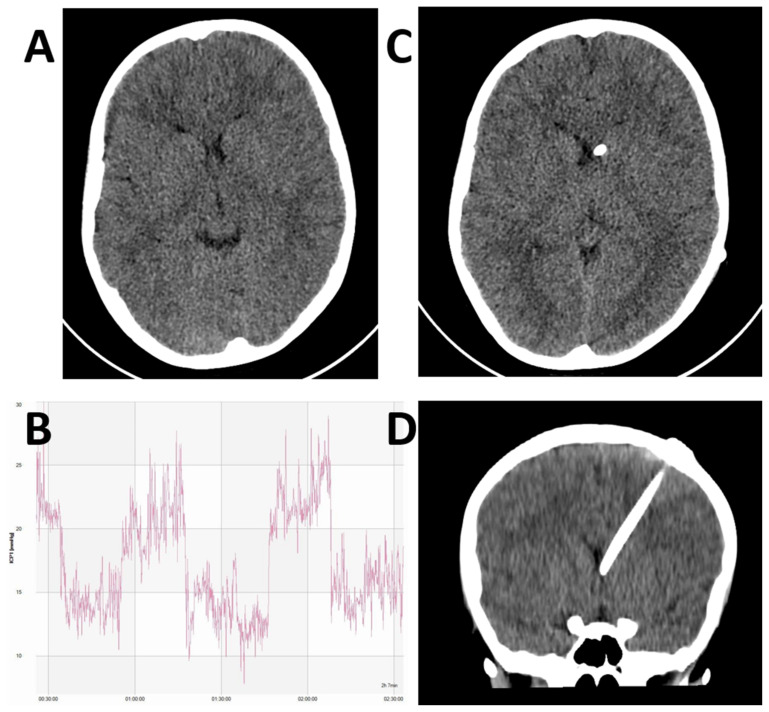
Illustrative case of a 9-year old boy presenting with chronic headaches and visual disturbance. Examination revealed bilateral papilloedema. The initial CT did not demonstrate a pathological mass lesion, but did show characteristic small ventricles (**A**). Lumbar puncture was equivocal with an opening pressure of 25 cmH_2_O. Pre-shunt invasive ICP monitoring demonstrated characteristic pathological plateau waveforms (**B**). A VP shunt was placed under EM guidance. Post-operative axial (**C**) and coronal (**D**) CT demonstrated satisfactory position of the ventricular catheter within the frontal horn of the left lateral ventricle.

**Figure 2:**
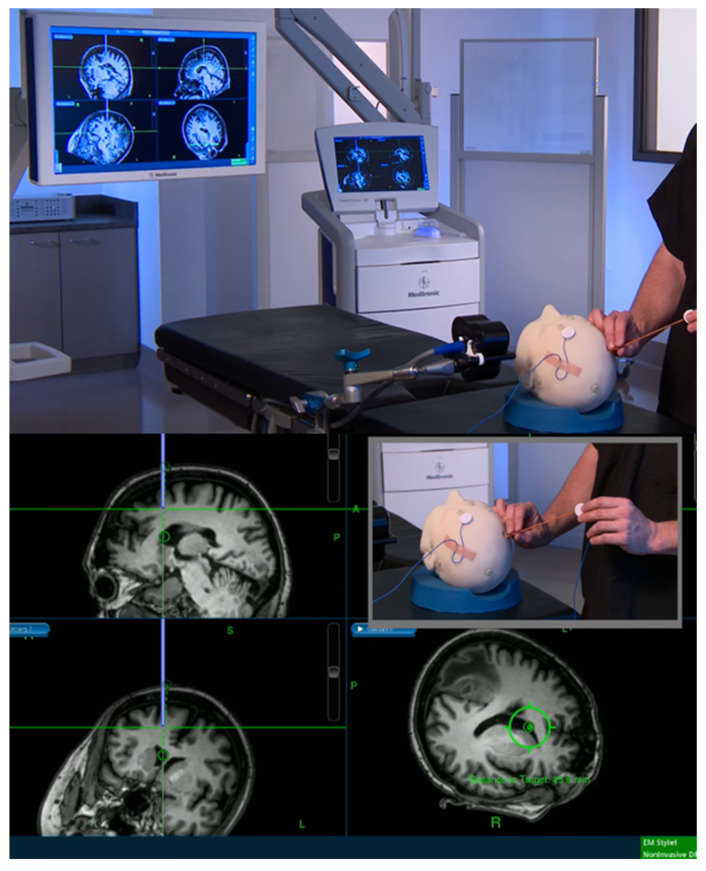
Demonstration of electromagnetic VP shunt insertion setup using the Medtronic AxiEM™ system. Screenshots from Medtronic promotional video.

The ventricles in IIH patients are not enlarged and typically may even be compressed (Figure 1) [41,45]. Accessing them, therefore, can present significant technical challenges. The advent and wide adoption of frameless stereotaxy systems within cranial neurosurgical practices significantly aid accurate placement of the ventricular catheter [41,46,47,48]. These systems provide an excellent level of accuracy, are routine to use, and do not substantially alter the operating time [49]. Electromagnetic guidance systems in particular have found favour due to their flexibility, ease of use, and avoidance of using pins to fix the patients’ heads in theatre (Figure 2) [50]. While they do not remove challenges of shunt placement in very small ventricles entirely, they aid and reassure the surgeon intra-operatively.

### 3.2. Complications

#### 3.2.1. Surgical

Serious complications associated with CSF diversion techniques are rare, although ventricular shunt placement is associated with more serious complications, including intracranial and intraventricular haemorrhage, infection, and CSF leak [51]. Furthermore, as an operation on the brain there is the risk, though very small, of developing post-operative epileptic seizures. In the UK, there is a statutory requirement for patients to inform the Driver and Vehicle Licensing Authority, who typically recommend a 6-month suspension from driving following VPS insertion. In addition to the intracranial complications, there are extremely rare, but potentially serious risks associated with tunnelling and placement of distal tubing, including vascular injury, pneumothorax, and bowel perforation [52]. The latter is a particular risk in patients who have had prior abdominal surgery and have intra-abdominal adhesions. Joint operating with general surgery colleagues or consideration of an alternative site of distal implantation is strongly advocated in these circumstances.

LP shunt placement is associated with a less serious operative risk as it avoids traversing neural tissue and, thus, the cranial risks of haemorrhage and seizures, and the potential restrictions on driving are negated. Over-drainage and development of secondary Chiari type I malformation are the most commonly reported complications [53]. Injury to neural structures in the caudal spinal canal, development of epidural haematoma, and cauda equina syndrome have the potential to cause permanent disability, though they are extremely rare. More commonly, patients are reported to experience radicular pain due to nerve irritation by the lumbar catheter. CSF leaks are also a potential consequence of LP shunt insertion, which can be problematic to treat.

#### 3.2.2. Shunt Failure

The vast majority of complications related to VP and LP shunts are mechanical failures and infections. Shunt revision rates overall are up to 25% within the first year and up to 85% within the patient’s lifetime [54,55,56]. Revision rates in LPS are particularly high, mechanical obstruction proving the major risk. In VPS, mechanical obstruction typically occurs as a consequence of shunt catheters lying in direct contact with the ependymal surface of small calibre ventricles. Some surgeons advocate for frontal placement of shunt catheters so that the tip lies free in the frontal horn of the lateral ventricle; however, there is insufficient evidence to support this practice [57]. It is also worth mentioning that elevated intra-abdominal pressure, as measured in overweight individuals, can compromise shunt function and limit the success of CSF diversion in patients who are not committed to weight loss as part of their overall management.

#### 3.2.3. Over-Drainage

Shunts may paradoxically cause complications due to over-drainage. Development of low-pressure symptom impacts on both VP and LP shunts though LP shunts are more severely affected due to the hydrostatic pressure effect of gravity on the fluid column within the neuroaxis in an upright position [58]. CSF leaks occurring around catheter insertion sites, particularly when shunts are inserted in an open technique, may further compound this [59]. CSF hypotension manifests symptomatically as low-pressure headaches, which are exacerbated by moving from recumbent to upright posture with associated nausea, unsteadiness, and even cognitive impairment [60]. Subdural haematomas may occur as a consequence of intracranial hypotension, as these ‘chronicify’, they can expand, exerting mass effect, and result in increased intracranial pressure [61]. Occasionally, these require surgical intervention to drain the collection.

The approach to management of over-draining shunts depends very much on the constituents of the implanted system. Conservative strategies include increasing the opening pressure of programmable valves where inserted, the addition of anti-siphon devices to shunt systems, or the use of gravitational valves. Disconnection and ‘tying-off’ of shunts can be performed, though this requires a further surgery to reconnect the shunt if needed at a later stage.

#### 3.2.4. Management of Suspected Shunt Malfunction, Obstruction, or Over-Drainage

**Figure 3:**
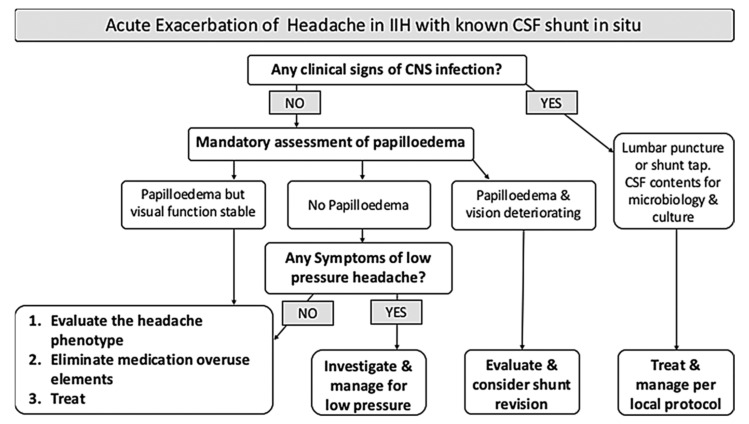
Algorithm outlining a systematic approach to assessment and management of IIH patients with VP shunts presenting with an exacerbation of headache symptoms. Adapted from Mollan et al 2018 [12]., used under creative commons CC BY 4.0 license.

The consensus guidelines on the management of IIH patients produced by the UK IIH special interest group produced a helpful algorithm for management of shunted IIH patients with headache exacerbation and suspected shunt malfunction (Figure 3) [12]. Patients with malfunctioning or obstructed shunts most commonly present with deterioration of headache symptoms and less frequently with acute visual disturbance [62]. Since headaches are not reliably improved in the long term by CSF diversion, this is not a particularly sensitive indicator of shunt failure. Attention is paid to visual symptoms and formal ophthalmological evaluation with dilated fundoscopy, VA, and VF assessment, mandated in any IIH patients with suspected shunt failure or malfunction [12]. Needle aspiration of the shunt reservoir is not especially useful in diagnosing shunt failure in IIH patients, as failure to aspirate more than a small volume of CSF more likely reflects small ventricular calibre and reduced intraventricular CSF volume, rather than proximal shunt obstruction. Nevertheless, obtaining CSF is crucial in the diagnosis of a suspected shunt infection and should be attempted in those presenting with systemic symptoms (fever, malaise), or with obvious meningism (headache, nausea/vomiting, photophobia, and neck stiffness). CSF obtained via lumbar puncture may be required and the opening pressure measured at this time can give valuable information regarding shunt patency. Cranial imaging is not a reliable indicator of shunt obstruction in IIH patients as ventricular configuration seldom changes, even in the context of shunt blockage [63]. Volume CT imaging may be required however in order to register for image guided shunt revision. Shunt series x-rays are useful to confirm continuity of the shunt system and can help adequately plan surgical intervention. They do not accurately predict shunt patency however [64]. CSF may continue to drain distally through a fractured or disconnected shunt, especially one that was implanted years ago, as a calcified tract may remain canalised.

Confidently excluding shunt malfunction or obstruction can be challenging in IIH patients, and surgical exploration to confirm patency or perform a shunt revision may be required, particularly if vision is threatened. As the neurosurgeon must be prepared to revise any or all components of the shunt, the patient is consented and prepped/draped in anticipation of a full shunt revision. Any hardware that may be required (proximal catheter, reservoir, valve, distal catheter) is brought to the theatre in case of need. In the majority of circumstances, only one component requires revision, most frequently the proximal catheter, followed by the valve, and rarely the distal catheter. Attempts should be made to explant failed components; however, this may prove difficult. Ventricular catheters commonly have choroid plexus adherent to them, which can bleed if avulsed. Tunnelled distal tubing that has been in situ for a number of years can be very difficult to extract, is occasionally disconnected, and left in place with new tubing connected to the functioning shunt. Malfunctioning valves should be replaced or correctly functioning valves exchanged for alternative models, e.g., with a higher opening pressure or a valve with a programmable facility in the context of over-drainage. The addition of an in-line anti-siphon device may also be considered.

### 3.3. Shunt Valve Selection

At their inception, the flow rate through CSF diversion systems was regulated simply by the resistance provided by the length of tubing, a slit valve at the very distal end, and by the pressure on the internal compartment the shunt drains to [65]. Since the development of the first widely used shunt valve, the Spitz-Holter valve, in the early 1950s, there have been countless technological developments, resulting in >290 valves brought to market. The design features and individual nuances have been comprehensively discussed elsewhere and this is beyond the scope of this article [66]. To this day, engineers continue to develop and refine technology to enhance shunt function and reliability, and new valve iterations are introduced regularly. There are numerous ways to categorise shunt valves, whether flow regulated or differential pressure controlled, programmable or non-programmable, with or without anti-siphon component, to name but a few key differences (Table 1). This complexity has confounded attempts to establish a hierarchy of shunt design and, subsequently, there is a lack of reliable evidence to compel the choice of one design over another.

#### 3.3.1. Flow Control vs. Differential Pressure

Non-programmable differential pressure (DP) valves can be considered as the standard CSF shunt valve. DP valves will open and permit flow of CSF once a threshold pressure (opening pressure) has been attained. This opening pressure in a DP valve is not an absolute pressure but rather the difference between inlet and outlet pressures. It holds then that transient increases in intracranial pressure or drops in outlet pressure (due to change in body position for example) can result in valve opening and drainage at lower relative intracranial pressures due to a ‘siphoning’ effect [67]. The rate of flow of CSF past this point is dependent on the pressure gradient, and this in turn can risk over-drainage and the development of intracranial hypotension, and even subdural haematomas. Flow regulated (FR) valves were developed to counteract this siphoning phenomenon and resulting over-drainage that is particularly problematic in idiopathic normal pressure hydrocephalus (iNPH) [68,69]. FR valves likewise have an opening pressure threshold but will regulate the rate of flow at a constant rate (mL/h), up to a critical level, above which free drainage is permitted as a safety feature.

#### 3.3.2. Programmable Valves

DP valves may come as fixed-pressure units with defined opening pressures, manufacturers typically providing a range of low, medium, and high opening pressure models to cater for the spectrum of hydrocephalic conditions. In addition, there are programmable models, which have an in-built adjustable component, which importantly can be altered post-implantation to fine-tune the CSF pressure/shunt flow dynamic, according to symptom control [70,71,72]. These valves are adjusted via application of a hand-held programmer which uses a magnetic device to ‘unlock’ and adjust the valve to its new pressure setting. The setting can then be confirmed via application of a reader device or by obtaining plain radiographs of the valve and comparing with a pictorial guide. The choice of one programmable valve over another very much depends on surgeon preference and the presence of other design features, such as anti-gravitational or anti-siphoning components. Pragmatic concerns, such as valve size and profile, as well as preferences surrounding the programming interface, also factor heavily. There is no definitive evidence to determine selection of one valve above a competitor. CSF drainage requirements can vary based on a variety of factors, fixed pressure shunt valves placed in paediatric patients for example may no longer provide the optimal CSF drainage as that child grows. IIH is a particular condition whereby the ability to adjust pressure setting can be useful. Balancing effective ICP management whilst avoiding over-drainage syndromes can prove challenging in some individuals.

#### 3.3.3. Anti-Siphon Devices and Gravitational Valves

In the closed (non-shunted) CSF compartment, CSF circulates from the site of production to reabsorption. An immediate drop in ICP is seen with change from recumbent to upright posture due to orthostatic changes in venous drainage. This drop is however attenuated, it is believed, by subsequent collapse of large draining neck veins [73]. The intracranial compartment is thus largely protected from large and sustained negative pressure conditions in the upright position [74]. Shunting between body compartments that are at different levels, e.g., head and abdomen will result in a differential pressure gradient, which will influence the flow of CSF within the shunt [75]. Flow within a CSF shunt is influenced by: (1) CSF pressure in the ventricle or lumbar cistern; (2) intraperitoneal pressure; and (3) hydrostatic pressure of the fluid column within the shunt [76]. Imbalance and unregulated flow leads to over-drainage and related conditions [75]. Programmable valves permit adjustment of the opening pressure; however, once adjusted that pressure is effectively fixed until any subsequent adjustment. A number of solutions to combat the issues related to orthostatic variations in CSF flow in shunts have been developed. In addition to the flow-regulated valves already discussed, standalone anti-siphon devices (ASD) exist, which can be installed in-line distal to the shunt valve [76]. Gravitational valves are a further alternative in which the siphon-reducing technology is integral to the valve mechanism itself and contained within the same housing [58]. Further to this, there exist programmable anti-siphon devices, which add a further level of complexity and versatility into the system.

### 3.4. Experience from the BASICS Trial

**Figure 4:**
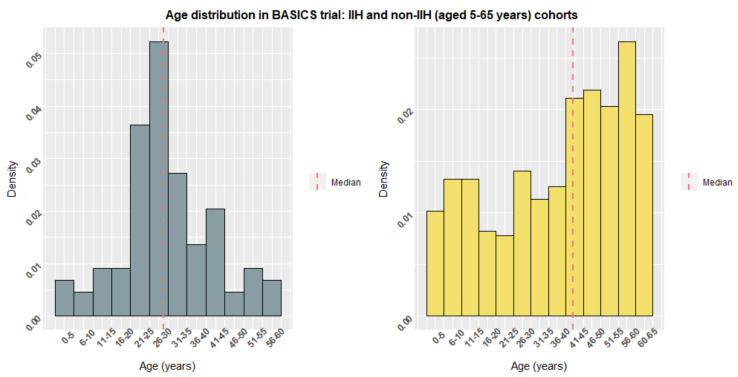
Density histogram demonstrating the age distribution of IIH patients compared with non-IIH cohorts (aged 5–65 years) from the BASICS trial.

The BASICS trial evaluated the efficacy of antibiotic impregnated catheters against standard and silver impregnated catheters establishing superiority of antibiotic catheters in reducing infection rates [44]. The trial enrolled 1605 patients of all ages and hydrocephalus aetiologies receiving de novo VPS insertion. The trial also enrolled patients with IIH and provided us with valuable data on CSF diversion and outcome over a 2-year follow up. Table 2 summarises the IIH cohort, including the valves implanted and revision rate, alongside an age-stratified cohort (5–65 years) of non-IIH aetiology patients for comparison. IIH patients had a lower median age, affecting most commonly females in their 3rd and 4th decades of life (Figure 4). All cause revision rates within 2 years were higher for VPS in IIH patients (32.9%) compared with non-IIH counterparts (23.6%). In addition, there was increased use of programmable valves and anti-siphon seen in the IIH cohort (Figure 2). Table 3 summarises the revision rate in IIH and non-IIH cohorts detailing modes of failure. Use of programmable valves and anti-siphon devices was associated with reduced revision rates. The BASICS trial was not designed specifically to assess the impact of valve/anti-siphon technology on shunt survival however, it provides 2-year follow up data for a large cohort of IIH patients with VPS. Interpretation of these data should be done with caution therefore, but they are nonetheless indicative of practices and outcomes across the UK.

## 4. Discussion

Idiopathic intracranial hypertension is a difficult condition to manage that can cause serious morbidity in otherwise healthy young adults if not adequately managed. Comprehensive guidance on the diagnosis and management of IIH has been produced, which emphasises the importance of a multidisciplinary approach [12]. CSF diversion is an important component of this strategy, but it serves only to mitigate the visual consequences of raised ICP and does not alter the underlying disease process. The most important and effective management strategy in the majority of cases remains weight loss. Active involvement of patients and their relatives is crucial therefore. Patients are relied on to be sensitive to subtle indicators of failing management, and to collaborate in regular ophthalmology check-ups. In the case of CSF diversion, this facilitates early identification, and effective intervention where a shunt is failing or malfunctioning. Patient and primary care education are thus a fundamental part of overall care.

While CSF diversion techniques are not reliable at controlling headache morbidity in the long-term, they are seen to improve symptoms in the short-term in the majority of patients. This can then serve as an adjunct to permit increased activity, positive change in mental state and result in sustainable weight loss. Over-drainage and the symptoms of low intracranial pressure can be problematic and difficult to manage. The evidence overall supports the use of VPS over LPS though a large recent retrospective analysis of 1082 shunts (347 LPS, 735 VPS) did however demonstrate no difference in revision rates [77]. This highlights the difficulties in evaluating comparative effectiveness based on data from largely retrospective case-series. Well-designed controlled trials are needed to evaluate the key questions regarding selection of shunt and valve type.

Insertion of a conduit into the CSF system that bypasses normal circulation will fundamentally alter CSF pressure dynamics. Some patients can be acutely sensitive to such changes though individual responses and tolerance of symptoms are not uniform or predictable. These challenges have driven development of valve technology in an attempt to recreate and maintain a physiological CSF pressure dynamic in shunted patients. There is comprehensive literature reporting on in-vitro performance of these technologies; however, evaluation in patients is difficult, not least because of the wide array of devices available [66]. The passage of CSF through a shunt is fairly predictable in an experimental setting, however, changes in posture, activity levels, CSF volume, viscosity, and fluid dynamics are just some of the unpredictable factors to consider in implanted shunts. A number of trials and reviews have sought to generate and evaluate evidence of superiority though these are not specific to IIH and while they tend to support the use of programmable valves and anti-siphon devices the evidence is far from conclusive [78].

Selection of specific valves and shunt technology should be done on a patient-by-patient basis. No one configuration has been demonstrated superior. Given the plethora of options available, management by a neurosurgeon with an interest in CSF disorders is advisable. These decisions should ideally be made in conjunction with the patient, explaining the features, advantages, and drawbacks of each option. It hardly bears mention that increasing complexity does not necessarily equate to superiority. Implantation of programmable components for example appears to offer only advantages. The programmable element can be immensely useful in certain circumstances, for example in managing symptoms of over-drainage. The programmable element can have the unwanted effect of increasing the number of consultations with anxious patients however, which has attendant impact on personnel and resources.

Implanted pressure monitoring devices can also be problematic. Evidence to support their use in IIH is limited [79]. In the authors’ collective experiences, they provide benefits, and can offer reassurance in the context of a normal reading or valuable warning of high ICP in a patient with a shunt obstruction. Readings can however be unreliable as sensors malfunction. They may also contribute to patient anxiety. More concerning are erroneous low readings, which may give false-reassurance. Treating clinicians need to maintain a high index of suspicion in a shunted patient presenting with worsening symptoms. Patient concerns over a potential malfunctioning or blocked shunt should not be dismissed and thorough clinical assessment including fundoscopy is essential. Excluding shunt obstruction is far from straightforward and may ultimately require shunt exploration. ICP monitoring provides very useful information and can be both diagnostic of a shunt obstruction or can guide proper selection of shunt valve and additional components. Neurosurgeons managing CSF disorders must become expert at interpreting the nuances of an ICP monitoring trace.

This review focused on CSF diversion strategies to manage the impact of IIH and deliberately avoids discussion of alternate surgical and interventional strategies, such as optic nerve sheath fenestration (ONSF) and intracranial sinus stenting. Both are viable and efficacious approaches with their own indications, advantages, and disadvantages [80]. There is currently a lack of data comparing ONSF, stenting, and CSF diversion, in terms of efficacy and longevity of effect [81]. Studies in this area would be of great value. Generating data on quality of life and neurocognitive outcomes following interventions for IIH in future studies would be particularly valuable as there is a notable paucity of such evidence in the literature.

## 5. Conclusions

CSF diversion constitutes the standard of practice for neurosurgical management of papilloedema and visual loss in IIH. It is safe and effective with both techniques; LPS and VPS showing equivalent and satisfactory efficacy. VPS has superseded LPS due to lower complication rates, in particular mechanical failure and over-drainage. Stereotactic (image guided) placement of ventricular catheters is strongly recommended in IIH. Increasing complexity of shunt valves available affords the surgeon many options to tailor shunt function to the patient’s needs. This requires a working knowledge of these devices, and as such, patients with IIH should be managed by a surgeon with an interest in CSF disorders.

## Figures and Tables

**Table 1:** Selected factors to consider when selecting valve technology in IIH patients.

Factors
Differential pressure vs. flow regulation
Fixed vs. programmable valves
Anti-siphon and programmable anti-siphon devices
Gravity assisted valves

**Table 2:** Number and age distribution of patients within the IIH cohort of the BASICS trial with patients from other aetiology cohorts between the ages of 5 and 65. The number and percentage of patients who had programmable valves implanted as well as anti-siphon devices (anti-siphon devices includes valves with integrated anti-siphon technology as well as separate anti-siphon devices) is also detailed.

	Idiopathic Intracranial Hypertension	Other Aetiologies
Total n	88	512
Female n(%)	78 (88.6)	271 (52.9)
Median Age (IQR)	31.2 (25.4–37)	44.6 (26.6–56.3)
Total Revisions n(%)	29 (32.9)	121 (23.6)
Programmable Valve n(%)	39 (44.3)	174 (34)
Anti-Siphon Device n(%)	31 (35.2)	139 (27.1)

**Table 3:** Number and proportion of VP shunt revisions including modality of failure (mechanical failure or infection) by whether the valve was programmable and whether an anti-siphon device was implanted (anti-siphon devices includes valves with integrated anti-siphon technology as well as separate anti-siphon devices).

	Revision	Mechanical Failure	Infection	Revision	Mechanical Failure	Infection
Total n (%)	29 (32.9)	24 (27.3)	5 (5.7)	121 (23.6)	101 (19.7)	20 (3.9)
Programmable Valve						
Yes n (%)	11 (28.2)	10 (25.6)	1 (2.6)	35 (20.1)	30 (17.2)	5 (2.9)
No n (%)	18 (36.7)	14 (28.5)	4 (8.1)	86 (25.4)	71 (21.0)	15 (4.4)
Anti-Siphon Device						
Yes n (%)	9 (29.0)	6 (19.4)	3 (9.7)	28 (20.1)	22 (15.8)	6 (4.3)
No n (%)	20 (35.0)	18 (31.6)	2 (3.5)	93 (24.9)	79 (21.2)	14 (3.7)

## Data Availability

Data from the BASICS trial is available on request from the original study investigators. Please see article for details.
